# Comparison of Mask-R-CNN and Thresholding-Based Segmentation for High-Throughput Phenotyping of Walnut Kernel Color

**DOI:** 10.3390/plants14213335

**Published:** 2025-10-31

**Authors:** Steven H. Lee, Sean McDowell, Charles Leslie, Kristina McCreery, Mason Earles, Patrick J. Brown

**Affiliations:** 1 Department of Plant Sciences, University of California Davis, One Shields Ave, Davis, CA 95616, USA; shle@ucdavis.edu (S.H.L.); caleslie@ucdavis.edu (C.L.); ktoporovskaya@ucdavis.edu (K.M.); 2 Viticulture and Enology, Biological and Agricultural Engineering, University of California Davis, One Shields Ave, Davis, CA 95616, USA; samcdowell@formerstudents.ucdavis.edu (S.M.); jmearles@ucdavis.edu (M.E.)

**Keywords:** machine learning, image analysis, computer vision, artificial intelligence, plant breeding, walnut, high throughput phenotyping, precision agriculture

## Abstract

High-throughput phenotyping has become essential for plant breeding programs, replacing traditional methods that rely on subjective scales influenced by human judgment. Machine learning (ML) computer vision systems have successfully used convolutional neural networks (CNNs) for image segmentation, providing greater flexibility than thresholding methods that may require carefully staged images. This study compares two quantitative image analysis methods, rule-based thresholding using the magick package in R and an instance-segmentation pipeline based on the widely used Mask-R-CNN architecture, and then compares the output of each to two different sets of human evaluations. Walnuts were collected over three years from over 3000 individual trees maintained by the UC Davis walnut breeding program. The resulting 90,961 kernels were placed into 100-cell trays and imaged using a 20-megapixel Basler camera with a Sony IMX183 sensor. Quantitative data from both image analysis methods were highly correlated for both lightness (L*; r^2^ = 0.997) and size (r^2^ = 0.984). The thresholding method required many manual adjustments to account for minor discrepancies in staging, while the CNN method was robust after a rapid initial training on only 13 images. The two human scoring methods were not highly correlated with the image analysis methods or with each other. Pixel classification provides data similar to human color assessments but offers greater consistency across different years. The thresholding approach offers flexibility and has been applied to other color-based phenotyping tasks, while the CNN approach can be adapted to images that are not perfectly staged and be retrained to quantify more subtle kernel characteristics such as spotting and shrivel.

## 1. Introduction

Consumer product preferences drive investment and research across many different crops [1,2], and the appearance of food plays an important role in consumer purchasing decisions. Of all visual traits considered by consumers, color is one of the most important. Color will drive consumers to a product [3], or away from it [4]. As a result, many plant breeding programs prioritize color as an important quality trait.

Walnuts (*Juglans regia*) were the ninth ranked agricultural commodity in California by value in 2022, at $1.24 billion [5]. The large majority of the more than 4000 walnut growers in the state are multi-generational family farms. Walnut kernels comprise an oil-rich embryo surrounded by maternal pericarp tissue commonly called the pellicle. The pellicle contains astringent phenolics that darken in response to oxidative stress, protecting polyunsaturated fatty acids in the embryo from oxidation [6]. Pellicle color is a key commercial quality indicator [7]. Nuts that are lighter in color command a higher price from processors and retailers, making pellicle color an important breeding trait [8]. Walnut pellicle color has traditionally been classified by color-matching to a USDA published chart of four categories: Extra-light, Light, Light Amber, and Amber. However, pellicle color is a complex quantitative trait with many environmental influences [9], and image-based phenotyping methods have been developed to quantify walnut pellicle color with higher precision and repeatability [10].

Computer vision involves teaching computers to recognize and analyze images like humans. Using a computer vision system (CVS) allows researchers to move away from manual grading, which is labor-intensive, time-consuming and suffers from inconsistent and inaccurate judgment [11]. Computer vision systems have been employed for agricultural uses since the mid-1980s and have relied mainly on thresholding-based techniques to classify objects in images. Thresholding methods involve setting specific pixel intensity values to separate objects of interest from the background. Initially, systems relied on monochrome images but quickly switched to color and hue-based methods [12]. While still used today, thresholding methods for image segmentation have fallen out of favor compared to machine learning methods, which use algorithms to learn patterns and features from large datasets, allowing for more complex and adaptive image classification.

Using machine learning in image segmentation can save time and costs compared to thresholding approaches [13]. The advantages of machine learning become more pronounced as the signal-to-noise ratio decreases or when there is high variability in image features, making traditional methods less effective. In greenhouse and field settings where the image background, placement of target objects, and lighting cannot be controlled, image segmentation is typically much more robust using machine-trained models compared to thresholding [14,15]. The primary disadvantage of using machine learning models is the large amount of human-labeled training data required to develop a robust model, even for relatively specific tasks. Training can typically be assisted using pre-trained datasets like Microsoft COCO [16], but these large datasets need more agricultural examples. Object detection can be more effective even with a slight modification to include agriculturally relevant pre-trained models [17,18].

A subset of conventional machine learning is deep learning, which learns to recognize patterns in data by looking at the information through a series of layers called neural networks. Combining the specific features of each layer produces a final algorithm that is used for higher-level phenotyping. CNNs (Convolutional Neural Networks) are a common class of deep learning algorithms typically used in image analyses due to their ability to identify spatial correlation among groups of pixels. Various computer vision tasks use CNNs, including image classification, object detection, and segmentation. CNNs have been successfully applied to measure leaf water status using both RGB [19] and multispectral imaging [20].

More recently, transformer-based vision models such as those using the Ultralytics YOLO framework have been rapidly advancing plant phenotyping tasks like leaf detection [21,22], and tomato defect segmentation and grading [23]. Vision transformers (ViT) often require larger annotated datasets and greater compute but are far better at capturing global contextualization through a self-attention process [24]. Hybrid systems combining both CNNs and ViTs can also leverage strengths of both approaches to collect local and global context for plant phenotyping tasks [25,26,27]. Multidisciplinary research leveraging AI and ML is increasing at an unprecedented rate [28], and in 2019, one deep-learning preprint was submitted to arXiv every 0.87 h [29].

The Mask-R-CNN architecture used for segmentation in this study can facilitate the training of objects that are far more complex than the staged images of walnut kernels. However, even in an almost perfectly controlled environment, there are situations where a deep learning model is advantageous for extracting pixel values of walnuts over a thresholding approach.

Most digital images contain data in the RGB color space, but other color spaces like CIELAB have been shown to better represent human vision [30]. The CIELAB color space, also referred to as the L*a*b* (where L* = luminance, and a* and b* are chromaticity coordinates) color space, was defined by the International Commission on Illumination in 1976. The commission designed the CIELAB color space to be perceptually uniform compared to an RGB-based color space, which aligns more closely with how human eyes perceive color differences. RGB color data records information on red, green, and blue combinations. However, when translating this data onto a screen for a human to perceive, every device can produce this image differently. Use of the L*a*b color space when analyzing color metrics is common in studies quantifying color data traditionally measured by humans [31,32,33]. New color spaces can sometimes even perform better than the established CIELAB color space [34]. Color data in the CIELAB color space is used in this study because it better reflects the human color perception of traditional grading. Additionally, the luminance channel correlates well with the four human scoring categories.

Developing a quantitative measurement system for grading color is important because it creates an unbiased system that consumers, growers, and researchers can all use. Human evaluation is inherently biased with inter- and intra- rater reliability, and inaccuracies with grading can slow down research, waste resources, and even impact grower profitability [35]. The importance of reliable and accurate measurements in plant phenotyping necessitates automated, consistent phenotyping systems that reduce bias and rater fatigue [36].

Currently, the DFA (Dried Fruit Association) of California serves as a neutral third party that can help facilitate fair evaluations of commodities, including walnuts, between growers and distributors. Companies like WECO (Woodside Electronics Corporation) have developed their Walnut Kernel Grader, which houses a camera system and screen to evaluate colors based on different grading scales. Amidst historically low prices and oversupply in the walnut industry [37,38], objective quality assessments ensure fair and consistent pricing of growers’ products. Many crops, including almond [39], cassava [40,41], and tomato [42], have already had objective RGB imaging systems developed for measuring color characteristics. All these imaging pipelines feature some use of either thresholding or machine learning approaches to process images depending on the complexity of each image.

Building on the growing importance of objective and precise image analysis in agriculture, the walnut computer vision system (CVS) presented in this study advances previous work to better meet the needs of modern breeding evaluations and quality assessment. Our approach is based on the CVS developed by Donis-Gonzáles for postharvest research [43]. This CVS was subsequently applied as a high-throughput phenotyping platform in a genetic analysis of kernel color in 528 trees from the UC Davis Walnut Improvement Program (WIP), identifying multiple small-effect QTL across seven chromosomes, and highlights the complexity of walnut kernel color [10]. In response to these challenges, this study enhances existing methodologies by (1) expanding the sample size to over 3000 trees, providing a more comprehensive genetic assessment; (2) improving image capture and analysis through a higher-resolution image sensor and a high-contrast background, leading to more accurate phenotyping; (3) systematically comparing two image processing methods—CNNs and traditional thresholding—to validate the robustness of our approach; and (4) implementing per-pixel color classification to generate a quantitative color score, offering a more precise and unbiased measurement of kernel color. This study not only refines image analysis techniques but also aligns modern machine learning methods with the needs of breeders, growers, and the walnut industry.

## 2. Results

### 2.1. Comparing Thresholding, CNN, and Human Vision

**Figure 1:**
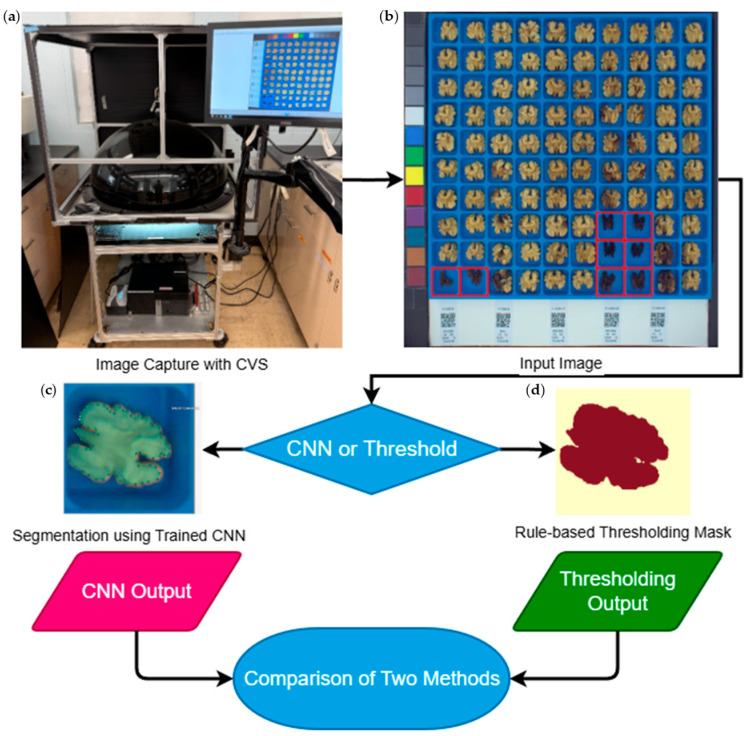
Flowchart of image and data processing. (**a**) Computer Vision System used for image capture, modified from [43]. (**b**) 100-cell blue tray raw image of ten-nut samples from five individual trees, with the two kernel halves of each individual walnut placed in adjacent columns. A color swatch on the left-hand side allows monitoring for consistent exposure and color between images. QR codes contain genotype and location information for each sample. Cells containing blank kernels are highlighted in red. (**c**) Computer Vision Annotation Tool (CVAT) annotation of an individual kernel using the polygon shape tool for use in the CNN training model. (**d**) Rasterized binary image of a kernel segmentation after thresholding out the blue tray using the magick package in R.

CNN and thresholding methods were used to process 930 images over 3 years (Table 1), which contained 90,961 kernels, along with 2152 blank kernels, from 4549 trees. “Blank” kernels, outlined with red in Figure 1b, are underdeveloped kernels that are shriveled, small, and dark. Blanks were manually scored in all images, however automated scoring performance from both methods is presented. Blank kernels are normally removed during commercial processing and are therefore not considered during breeding program color evaluations. Blanks were removed from the dataset before downstream color processing.

Table S1 contains all output from both CNN and thresholding methods, including the 10th, 25th, 50th, 75th, and 90th percentiles and average values of L*a*b* and RGB from every kernel processed, as well as the size in pixels of each kernel. Since the human color scores were graded on a per kernel basis (two halves together), quantitative data were summarized by taking the average of the two halves of each kernel.

**Figure 2:**
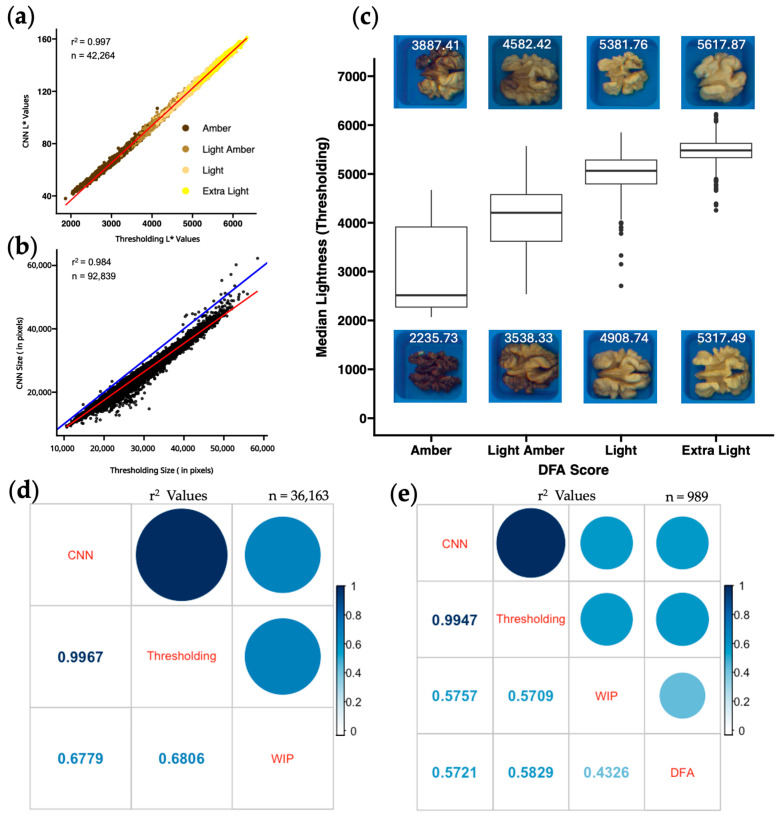
Comparison of computer and human walnut kernel phenotypes. (**a**) Median L* values on a per-nut basis measured with CNN and thresholding with the fitted linear regression line in red (r^2^ = 0.997). The color of each point represents the WIP human color score. (**b**) Size (in pixels) on a per-kernel basis measured by the CNN and thresholding methods with the fitted linear regression line in red (r^2^ = 0.984) and a line of perfect agreement (y = x) in blue. (**c**) Boxplots of median L* values from the thresholding method on a per-kernel basis for each human category in the DFA dataset. Kernel images above and below each boxplot represent kernels at the first and third quartiles of median L* for that DFA color score. (**d**,**e**) Coefficient of determination (r^2^) matrices comparing median L* values from two computer methods (CNN and thresholding) to two human methods (WIP and DFA) on a per-nut basis. The DFA dataset is much smaller than the other datasets.

Overall, the two methods strongly agree for median lightness (r^2^ = 0.997; Figure 2a) and kernel size in pixels (r^2^= 0.984; Figure 2b). Comparison of the red trendline and blue identity line (y = x) in Figure 2b shows that kernel size is consistently smaller with the CNN method, likely because the manual annotation of kernels in the CNN training set tended to exclude pixels at the periphery of the kernel (Figure 1c). The mean difference between the two is 3441.69 pixels, or 12.02%.

Points in Figure 2a are colored by their human color scores, scored by a WIP team member under fluorescent lights in a laboratory. In order to determine which color value is most related to how humans grade kernels, linear regressions were performed between human color scoring and all the different percentiles of L*a*b* and RGB. Color scores for individual kernel halves were converted to a numeric scale (1 for amber, 2 for light amber, 3 for light, and 4 for extra light). Median L* from both thresholding and CNN methods explains roughly 70% of the variance in human color score, r^2^ = 0.681 for thresholding and 0.678 for CNN (Figure 2d).

In 2021, a subset of 2000 kernel halves was sent to the California DFA to obtain a neutral third-party evaluation of kernel color, graded by a human observer under fluorescent lights in a warehouse. The resulting scores were compared against the median L* from the thresholding method (Figure 2c). Images above and below each box in the boxplot are individual kernels with L* values corresponding to the first and third quartiles of each color category. Amber-graded kernels have the most variability in median L* values, while extra light-graded kernels have the least variability. Sample size was much smaller in the DFA subset, and the WIP human color scores had much higher correlations with median L* (r^2^ = 0.7 compared to 0.54 in the DFA), with only modest correlation between the two human color scores (DFA and WIP, r^2^ = 0.4 (Figure 2e)).

### 2.2. Lightness (L*) Color Thresholds

**Figure 3:**
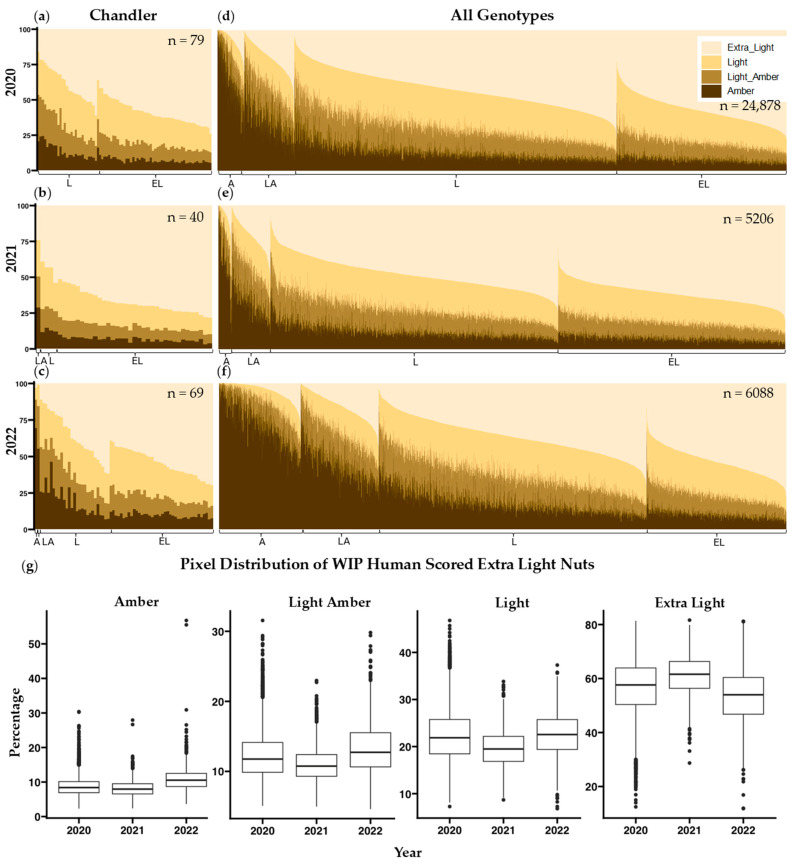
Pixel classifications on a per-nut basis of the cultivar “Chandler” (**a**–**c**) and all genotypes (**d**–**f**) over 3 years in 2020 (**a**,**d**), 2021 (**b**,**e**), and 2022 (**c**,**f**). Each vertical bar represents a nut. Nuts are arranged by their WIP human color score (EL, L, LA, and A) as shown on the x-axis, while the y-axis shows the percentage of amber, light amber, light, and extra light pixels. (**g**) Year-to-year variation in pixel classification of all samples scored by humans as “extra light.”

Using a linear regression between ordinal human color scores and median L* from the thresholding method, predicted L* values at 1.5, 2.5, and 3.5 (halfway between each color score) were obtained and used as thresholds between color categories (Figure S1). Pixels with L* values greater than 5292.13 are categorized as extra light, pixels between 4610.63 and 5292.14 are light, pixels between 3929.14 and 4610.63 are light amber and pixels below 3929.14 are amber. Comparison of pixel classification results with human scores for individual nuts shows both misclassification of kernels (for example, a kernel scored as extra light (EL) with lower lightness (L*) than a kernel scored as light (L); (Figure 3a–f), as well as year-to-year variation in the human assessments (Figure 3g).

### 2.3. Blank Kernels

The two methods handled blank kernels differently. For the CNN method, blank kernels were intentionally left unannotated in the training set. For the thresholding method, generalized linear regression using the GLMNET package in R was used to predict blanks using size and color. Since blank kernels are almost always smaller and darker than normal non-blanks, size and lightness were good predictors. The final predictors included in the model were kernel size, 10th percentile of L*, mean red, and mean blue. All 52,718 kernels harvested in 2020 served as the observations used to build the model, which was then used to predict blank kernels in 2021 and 2022.

**Figure 4:**
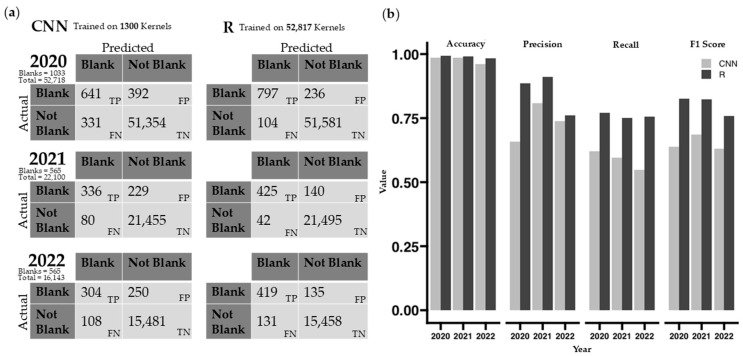
Performance metrics for automated detection of “blank” kernels. (**a**) Confusion matrices for both CNN and thresholding models for blank detection. (**b**) Accuracy, precision, recall, and F1 performance metrics for CNN and thresholding methods of automated blank detection. Blank kernels are the positive class used for calculations. The CNN method was only trained on 1300 kernels, compared to 52,817 kernels for the thresholding method.

The performance of the two methods for automatically removing blank kernels was assessed using accuracy, precision, recall, and F1 score (Figure 4). The GLM (generalized linear model) in R outperformed the CNN method in all metrics, especially in precision, recall, and the F1 score. However, CNN detection only had 2% of the training kernels that the GLM had. Both methods maintained a high accuracy for all three years.

## 3. Discussion

This study details the advantages and disadvantages of a simpler thresholding-based approach to a more complex machine learning approach of measuring walnut kernel color, while highlighting quantifiable differences. The WIP has historically evaluated kernel color using human raters, and an objective method to quantify kernel color was only recently developed [10,43]. The decision to fully embrace a new method of grading necessitates detailed documentation of methods as well as transparency in development and implementation. While there will often be newer and faster software, especially in the growing field of AI/ML, it is important to benchmark newer methods against established ones.

The use of image-based phenotyping has been shown to be more effective than human grading [44]. Agricultural products like apple [45,46], banana [47], blueberry [48], and oranges [49] have image-based grading systems that outperform human scoring. Our findings suggest the same: human evaluation of walnut kernel color is inconsistent, with limited agreement between human scorers (r^2^ = 0.433; Figure 2e), whereas two image-based methods produce nearly identical results (r^2^ = 0.997; Figure 2d). The 90,961 kernels from the 3000 unique trees used in our analysis provide the largest multi-year dataset of its kind. With a genetically diverse phenotypic dataset collected across multiple environments and years, we are confident in the ability of both methods to consistently output repeatable walnut kernel color data from photos taken with this CVS.

### 3.1. Challenges with Shelling and Image Capture

While manual kernel shelling results in mostly intact halves, some kernels end up in fragments. Consequently, when these fragments are included in the analysis, they can lead to inaccurate color measurements as the interior embryo is exposed. While this issue affects only a small proportion of the samples, it impacts the measurements’ overall accuracy. Because there is little color difference between the interior embryo and an extra-light pellicle, the thresholding method would not likely be able to correct this issue. In contrast, the CNN could specifically be trained on images that included kernel fragments, with portions of exposed embryo left unmasked. A YOLOv5 based model has already been developed to sort and grade kernels based on fragmentation [50].

Another notable challenge was handling the presence of overlap between exceptionally large kernels in the trays. While overlapping kernels were intentionally included in the CNN training set, the thresholding approach is more rigid. It may occasionally include part of one kernel in another kernel’s measurement, though overlapping kernels are always samples from the same tree.

Image capture resulted in barrel-distorted photos, also known as the fisheye effect, due to the 8 mm focal length non-rectilinear wide-angle lens. Using version 6.0.13.7126 of Pylon Viewer, there was no option to correct barrel distortion natively, and images had to be post-processed in order to preserve accuracy between inner and outer kernels in the image.

### 3.2. Challenges with CNN

The CNN method’s effectiveness relies on representative samples in the training set to avoid challenges like model overfitting and generalizations. The CNN used in this study performed very well despite the inclusion of just five images in the training dataset, four images in the test dataset, and four images in the validation dataset. Annotated images included kernels of varying shape, color, and size. Training the model requires higher computational resources than thresholding. Adding more images to any of the three training datasets will increase the computational time but provide a more robust model [51]. Processing these images is also cumbersome if running locally on a consumer machine not optimized for Pytorch. Google Colab (https://colab.research.google.com/) makes it convenient to run machine learning pipelines, as libraries like PyTorch and TensorFlow have already been pre-installed. Google Colab also provides free limited access to optimized GPUs and TPUs for these workflows.

### 3.3. Challenges with Thresholding

Creating the thresholding script revealed unanticipated challenges that required substantial manual iteration which is challenging for automation and scaling. Finding a color threshold to segment each kernel from the background took a few manual iterations; however, incorporating red and blue values in the threshold calculation was robust enough to separate the kernel from the tray. The next challenge involved defining a kernel cell coordinate system that was stable enough to define all 100 kernel cells in every image accurately. Despite having a positioning guard when staging every photo, there was always some degree of rotation that offset every image slightly. By cropping out all parts besides the blue tray in every image and applying an image rotation function, the coordinates were only measured once and then used for all other images.

Another challenge was dealing with small pieces of dust or debris that were present in some kernel cells. A size threshold of 500 pixels was sufficient to separate dust and debris from true kernels. These challenges highlight the extensive process of quality checking the thresholding pipeline, with a lot of time spent updating the code to handle all situations through trial and error.

### 3.4. CNN vs. Thresholding

The CNN and thresholding methods provided similar results in most color categories despite being calculated on different scales. Data processed through either method would be sufficient for future projects that need accurate and consistent measurements of pellicle color. Each method had its drawbacks. However, the advantages of each method are clear. Training a CNN model to identify and segment out walnut kernels in an image provides a more robust method that can be more adaptable to a change in condition. Rotational inconsistencies were not an issue for the CNN method, and this method should be more tolerant to potential future changes in imaging conditions like lighting, positioning, and image quality.

Conversely, while the thresholding method may not be as robust and adaptable as the CNN approach, with the relatively stable conditions of the staged photos, the thresholding process is more intuitive and less resource-intensive to run. Thresholding provides the same data more quickly and easily. Although Google Colab resources are openly available to the public, there are daily processing limits, so the CNN method required several days to complete. These resource requirements, however, continue to decrease as an increasing number of CNN models can be run on consumer-grade hardware. R and the magick package are free, open-source programs that can more easily be run on consumer computers.

### 3.5. Blank Kernel Detection

Blank kernels result from incomplete kernel filling due to physiological stress and are outliers for pellicle color. Different stages of shrivel [52] have been classified in previous research. Blanks in this study were manually flagged and removed during data processing but automating blank removal using either method could make this process even more efficient. The comparison between the two automatic blank removal methods is biased in the number of kernels in each training set. The CNN method only used 13 images to generate a model, totaling 1300 kernels, whereas the GLM in R utilized all 52,817 kernels from 538 images taken in 2020 for its training model. The difficulty in this comparison is that the CNN requires training before data processing, while the GLM uses acquired data to make a model after data image processing. Both models can be improved with more kernels used for training.

In our dataset, non-blank kernels (97.6%) far exceed the number of blank kernels (2.4%; Figure 4b). Performance metrics, particularly accuracy, are heavily skewed due to the imbalanced dataset. However, considerably higher recall, precision, and F1 scores show that the GLM model is better at both capturing true positives (blanks) and handling false positives and false negatives (Figure 4a).

### 3.6. Comparing Quantitative Color to Human Color Scoring

Human color scores produced by two different groups of trained observers (WIP and DFA) did not agree well with each other or with either of the computer vision scores (Figure 2e). This result agrees with previous work showing variability in human ratings [35,36]. A stepwise regression analysis in R, additional predictor variables, and a single-color phenotype did not yield significantly better r^2^ values when comparing computer vision data to human scored data. Similarly, a principal component analysis resulted in no components that explained greater than 80% of the variance. Our approach was to establish L* thresholds between human color categories and present the percentages of each color category per sample. This method of presenting color values of individual pixels is also used by the Walnut Kernel Analyzer, a product manufactured by WECO that has gained popularity among California walnut processors. Samples were assessed using this method revealing misclassifications in scoring (Figure 3d–f) and a considerable amount of variability in human scoring between years (Figure 3g). Human assessment of a given sample is likely influenced by external factors such as preceding samples evaluated, and other neighboring samples on the same tray [53]. Figure S2 shows distributions of pixel category classification for all four-color categories distributed across the three years and a Tukey multiple pairwise comparison test shows that all but 4 of the 48 total pairwise comparisons per human color classification are different (*p* < 0.01). Consistency between trained observers in the WIP and the DFA is also very low (r^2^ = 0.43 Figure 2e). The reliance on human scoring is detrimental to current growers, because their profits rely on subjective grading. In contrast, the correlation between image-based phenotyping methods for kernel color remains very high (r^2^ > 0.99) across all three years of our study (Figure S3), suggesting that both these methods can be applied to future years without recalibration.

## 4. Materials and Methods

Figure S4 in the Supplementary Materials provides a more detailed flow chart describing the data processing steps.

### 4.1. Walnut Harvest and Shelling

Mature nuts from *Juglans regia* trees were harvested into plastic mesh bags between August and October 2020, 2021, and 2022. A random sample of at least 20 nuts was hand-harvested from each tree when the outer pericarp (hull) could be cleanly separated from the inner endocarp (shell). Nuts were air-dried before storage at 4 °C for up to 6 months before being shelled.

During shelling, kernel halves from a random sample of 10 nuts from each sample were carefully extracted using a hammer, a dull knife, and a wooden block. After shelling each nut, the two kernel halves were placed in adjacent columns of a blue 100-cell tray (10 columns × 10 rows). Trays sourced from the DFA were spray painted “Oasis Blue” using Rust-oleum 2X Ultra Cover Paint + Primer spray cans (Rust-Oleum, Vernon Hills, IL, USA). The 10 kernels (20 halves) from a single tree/bag filled exactly two columns and ten rows. Stickers with QR codes encoding the genotype and location of the tree were placed at the bottom of columns 1, 3, 5, 7, and 9 to label each sample (Figure 1b).

### 4.2. Manual Color Grading

Kernel color was graded on a four-point scale (from darkest to lightest: Amber, Light Amber, Light, and Extra Light) using the standard DFA color chart (Figure S5). A single trained individual scored kernel color every year under fluorescent lights on a lab bench with variable additional natural light. A subset of 2000 kernels from the 2021 harvest was sent to the commodity inspection department of the California DFA to receive their expert evaluation.

### 4.3. Image Acquisition and Pre-Processing

Using the image capture box developed by Donis-Gonzáles, 100 kernel halves were placed embryo-side down in 390 × 390 mm blue trays with color values of 33–57, 72–120, and 106–191 for R, G, and B, respectively. The highest values represent the face of the trays with no shadows, and the darker values represent the inner walls of each cell, where the color is slightly darker. The hex code of the lightest blue color is #21486a, and the darkest blue is #3978bf.

The camera system consists of a Basler acA5472-5gc GigE camera with a 1-inch Sony IMX183 CMOS sensor (Basler AG, Ahrensburg, Germany) delivering 5 frames per second at 20.0 MP resolution and fitted with a Kowa LM8HC f/1.4–f/16 aperture, 8 mm fixed focal length lens (Kowa American Corporation, Torrance, CA, USA). It is mounted on top of a MB-DL906 (Metaphase Technologies, Inc., Bristol, PA, USA) diffused dome light controlled by a ULC-2 universal LED controller (Metaphase Technologies, Inc., Bristol, PA, USA). Underneath the camera is a sliding tray sized to fit the 100-well kernel trays. Captured JPEG images used a white balance setting of 5000 K, f/1.4 aperture, and a shutter speed of 1/125 of a second. Additional color parameters used for image capture can be loaded into the software via the .pfs file (File S1). Every image captured retains the exposure and color settings written in the .pfs file. The color swatch included in each photo was also checked for consistency between sessions for all data collected.

The distance between the tray and the camera is 430 mm. Black materials are used to minimize reflectance onto the target kernels. The camera is connected via a CAT5 adapter to a Dell OptiPlex 7040 running Windows 10 Enterprise 2016 LTSB. Using 64-bit PylonViewer software version 6.0.13.7126 individuals can operate and capture a photo with a resolution of 3300 × 3120 pixels. Combining a consistent lighting scheme, enclosure setup, and camera settings allows for equal comparisons between different photos. Figure 2 outlines the pipeline, beginning with image capture.

### 4.4. Lens Correction/Image Adjustment

Due to the wide-angle 8 mm focal distance lens used, barrel distortion, also known as the fisheye effect, is seen in the captured images. This distortion is a common optical aberration that results in a curved representation of objects, typically affecting objects near the edges of the image. Lens distortion does not affect the color intensity of the image but can affect the geometry of objects in the photos, depending on the location within each photo [54]. The image_distort function in the magick package was used to correct the lens distortion using a barrel lens correction of 0, −0.22, 0. After applying this function, tray edges and kernel geometry appear rectilinear. This is confirmed by the final 2780 × 2780 cropping of only the blue tray that is used in the thresholding script. The square crop of the tray and the square crop of each well rely on no distortion being present.

### 4.5. Blank Kernel Removal

The number of blank kernels in a sample is recorded because repeated incidence of blanks can indicate physiological problems with a tree. However, blank kernels are excluded from kernel color datasets before analysis because they are not physiologically mature, and their color and size do not reflect the genetic potential of a tree. Automatic detection and removal of blanks would save the time currently required to label blanks manually. When annotating images for training of the CNN model, blanks were intentionally left unannotated.

### 4.6. CNN Method

The custom model developed for this study was built using the widely adopted Mask-R-CNN architecture with a ResNet-50 + FPN backbone. We implement the reference TorchVision model and re-initialize the detection and mask heads for two classes (background, walnut), then train the weights on our annotated images. 

The software used for this processing can be downloaded at:

www.github.com/DigitalAgSL/walnutpheno (accessed on 24 October 2025)

Additional Resources for machine learning in agriculture can be found here:

www.github.com/project-agml (accessed on 24 October 2025)

#### 4.6.1. CVAT Annotation

The first step in this method is to use the Computer Vision Annotation Tool (CVAT) software to annotate the kernel images. The thirteen images selected for annotation included examples of blank kernels (not annotated), overlapping kernels, broken kernels, light kernels, and dark kernels. The polygon shape tool was used to create masked kernels for annotation labeling; however this process was time consuming; each image took roughly 20 min to complete. More recently, automatic segmentation methods using Meta AI’s Segment Anything [55] can reduce this annotation time to about a minute an image. The patience and precision of the human annotator creating the training set is a potentially limiting factor in the CNN method. Our manual annotation erred on the side of excluding pixels at the edge of each kernel to ensure that no blue pixels were included in the masks.

#### 4.6.2. Model Training

The next step is to train the model using the annotated images, which are separated into a training set, a test set, and a validation set. Of the thirteen annotated images, five images were used for training, four images were used for validation, and four images were used for testing. The uses of these three datasets are described in Pattern Recognition and Neural Networks [56] as follows:

- Training set: A set of examples used for learning, that is, to fit the parameters of the classifier.
- Validation set: A set of examples used to tune the parameters of a classifier, for example, to choose the number of hidden units in a neural network.
- Test set: A set of examples used only to assess the performance of a fully specified classifier.

#### 4.6.3. Mask-R-CNN Image Processing

Once a model was developed from this training, the CNN_detection python script was run through the free version of Google Colab for inference. Input images are placed in the walnut_images folder on Google Drive. Google Colab provides a free and easy way to run PyTorch code, as it has the required packages and dependencies installed in the working environment. After the images are uploaded to Google Drive, the script can be run with minimal changes to the code, the only change needs to be renaming the output file.

Using the trained model, kernels are identified and iteratively processed, recording the 10th, 25th, 50th, 75th, and 90th percentiles of R, G, B, and L, A, B, values along with the size in number of pixels. The output CSV file is then generated in the wlnt_detection_results folder on Google Drive. The output file is in a long format, with each data point in a row. An R script is used to convert the long format to a wide format where the RGB, L*a*b*, and size values are put into columns for each kernel.

### 4.7. Thresholding Method

The magick package in R includes all tools in the ImageMagick STL open-source image processing library used by the thresholding method. Image files are imported into the R environment with the image_read function and saved as external pointers of the class ‘magick-image.’ File S2 contains the R code to process images, and File S3 loads coordinates of the 100 cells in the tray. These can also be found in the GitHub repository.

#### 4.7.1. Image Cropping, Rotation, and Coordinates

The imaging system was designed to ensure consistent positioning of kernel trays within captured images. However, because the thresholding method assumes that each kernel cell will occur at fixed coordinates within each image, slight mispositioning of the tray can cause problems where portions of non-targeted kernels are included, or portions of targeted kernels are excluded from a given kernel cell. In the early stages of development of the thresholding method, comparison with data from the CNN method identified slight inconsistencies in the vertical alignment and rotation of the tray that necessitated the addition of code to rotate and crop each image. The first loop in the thresholding script scans the top and bottom 20% of each image and using the mean red and blue values as a binary threshold, determines where the left edge of the blue tray begins at the top and bottom of each image. Then, the image_rotate function is applied, using the degree of rotation calculated by the positional difference in pixels between the top left and bottom left edges of the tray.

After the image is rotated, a binary color threshold is again used to find the edges of the blue kernel tray in the newly rotated image. Using the mean red and blue values of every single row (3300 pixels) and column (3120 pixels) in the image, the program iteratively processes top to bottom and then left to right to determine where the blue tray starts and everything else ends, including the color swatches, white QR-coded labels, and black background. This process produces a 2780 × 2780-pixel final image containing only the blue tray. Kernel cell coordinates were manually defined a single time using ImageJ v1.53 and the rectangle tool to draw a 270 × 270-pixel box over each cell. Cell coordinates are defined with respect to the (0,0) pixel at the top left of the tray.

#### 4.7.2. Magick Image Processing

Once images are standardized, the script iteratively processes through each cell in the tray, segmenting out the tray from the kernel using a difference of r_values and b_values. The tray is defined as all pixels for which r-b ≤ −40. All other pixels within this cell are defined as a kernel. To exclude empty cells with small fragments of kernel material or other debris, at least 500 kernel pixels are required per cell to be considered a true kernel. The inner loop iteratively processes through each of the 100 kernel cells and records the average RGB and L*a*b* values of kernel pixels as cell as the number of pixels classified as kernel. The 10th, 25th, 50th, 75th, and 90th percentiles of RGB and L*a*b* values are also recorded, to match the output of the CNN method. The outer loop processes all the photos in the directory.

#### 4.7.3. QR Detection and Method Comparisons

After obtaining color and size data on individual kernel halves from the CNN and thresholding methods, data are grouped by sample using the qr_scan function to record the text embedded in each QR code and attach it to the corresponding sample. Data are handled using “R” software v4.03 with packages “tidyverse”, “corrplot”, and “ggplot2”.

## 5. Conclusions

Both CNN and thresholding methods are suitable for obtaining consistent quantitative color data from walnut kernels. Deciding which method to use depends on the size and complexity of the dataset, access to computing resources, and how much training is required. The advantages of the CNN method are its robustness to changing variables and its efficiency in development. Although staged images were used in this study, slight variations in the placement of kernel trays resulted in a long and tedious process of corrections and adjustments to the R thresholding code. However, once fully adapted to these inconsistencies in tray positioning, the thresholding method produces the same data as the CNN method and uses less processing power.

The thresholding method required substantial tuning to compensate for varying conditions, even in these staged images. While the development took longer than the CNN method, our final thresholding pipeline offers easier adaptability to phenotype anything that can fit into the 100-well trays. One example is adapting the thresholding script to quantify the infection of walnut hulls by the walnut blight pathogen (*Xanthomonas campestris* pv. *juglandis*), which causes black lesions; this only required changing a few lines of code. To accomplish this via ML would require training a new model, which would be slower.

The study was conducted using carefully staged images, requiring careful uniform placement of walnut kernels. This represents a “best-case scenario” for thresholding. Additional work is needed to explore how these methods could be applied in industrial or commercial conditions. Implementation of a newer ML model seems like the ideal path forward, especially considering advancements in AI and newer ViT architectures like YOLO and RT-DETR. Using newer models alongside multi-class segmentation, we can potentially expand our capabilities and train a model to quantify other visual phenotypes such as pellicle spotting, kernel shrivel, and fragmented kernels. These modifications would enable further increases in both the efficiency of walnut breeding and the fairness and transparency of the commercial walnut grading process.

## Figures and Tables

**Table 1:** Summary of images processed.

Year	Images	Kernels	Trees	Blank Kernels
2020	538	52,718	2641	1033
2021	226	22,100	1100	565
2022	166	16,143	808	554
**Total**	**930**	**90,961**	**4549**	**2152**

## Data Availability

The dataset supporting the conclusions of the article is included within the article (and Supplementary Materials). The CNN script is made available through www.github.com/DigitalAgSL/walnutpheno (accessed on 24 October 2025).

## Supplementary Materials

The following supporting information can be downloaded at: https://www.mdpi.com/article/10.3390/plants14213335/s1, Figure S1: Linear regression between median L* from thresholding and human color score; Table S1: 92,839 × 72 table containing all the color data from both CNN and thresholding methods; Figure S2: Pixel distributions of WIP human scored nuts for all four classifications; Figure S3: Correlation matrices between both methods and human scores, separated across three years; Figure S4: Detailed flow chart describing the data processing steps; Figure S5: USDA Walnut Color Chart used by the California Dried Fruit Association; File S1: Features file to be loaded into Pylon Viewer to adjust color capture settings; File S2: Thresholding script in R; File S3: Coordinates file for thresholding script.

## Abbreviations

The following abbreviations are used in this manuscript:

CNN	Convolutional Neural Network
CVS	Computer Vision System
RGB	Red, Green, Blue
CIE	Commission on Illumination
L*	Luminance
a*	Red / Green chromaticity
b*	Blue / Yellow chromaticity
DFA	Dried Fruit Association
WIP	UC Davis Walnut Improvement Program
GLM	General Linear Model
CVAT	Computer Vision Annotation Tool
ViT	Vision Transformer
