# Supplementary material for: Comparison of Mask-R-CNN and Thresholding-Based Segmentation for High-Throughput Phenotyping of Walnut Kernel Color
Source: Plants (Basel). 2025 Oct 31;14(21):3335. doi: 10.3390/plants14213335 (PMC12610562; doi:10.3390/plants14213335)
Supplement: Supplementary file 1 [file plants-14-03335-s001.zip › plants-3922229-supplementary_rev1/Figure S2.pdf]

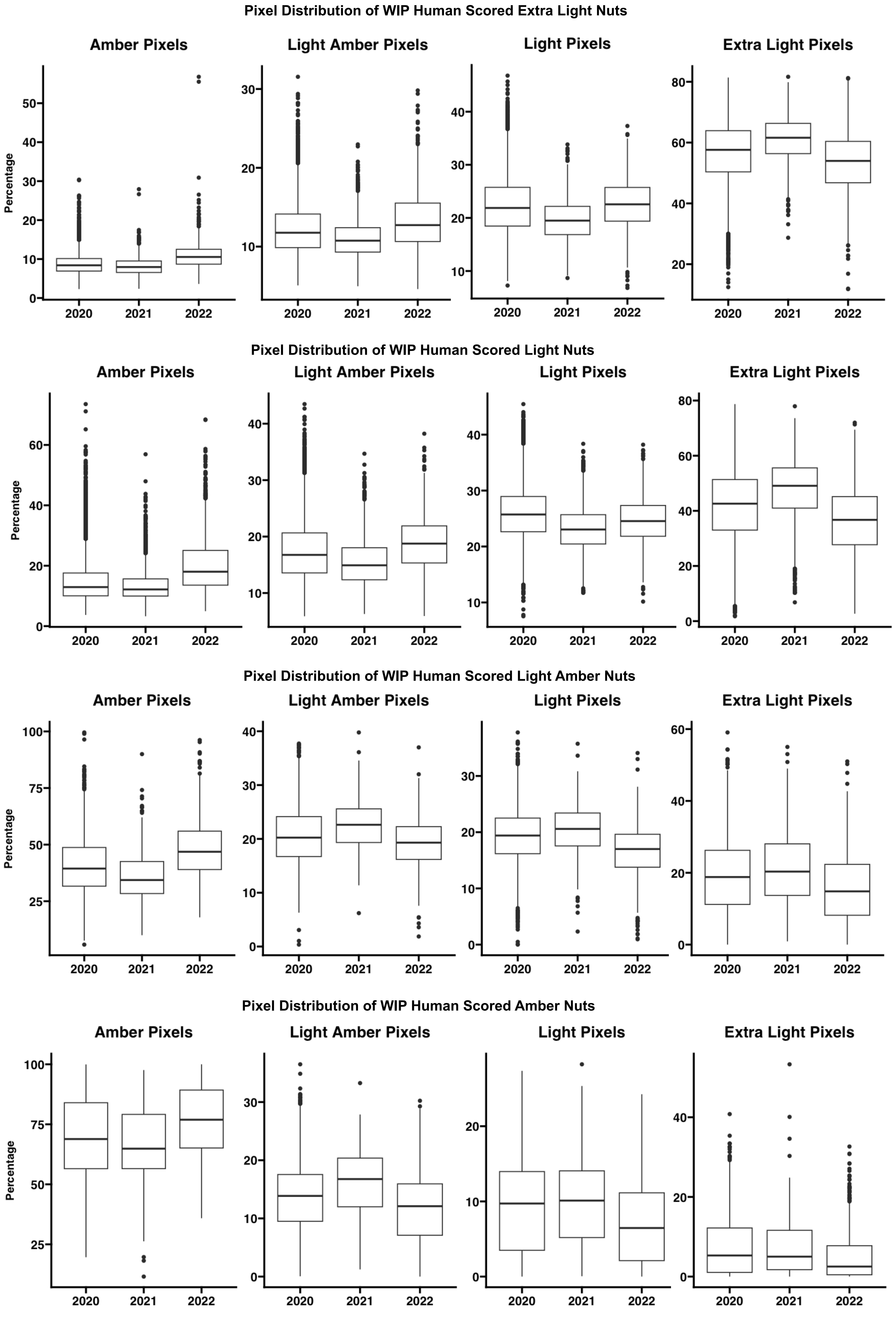
Pixel Distribution of WIP Human Scored Extra Light Nuts
Amber Pixels
Light Amber Pixels
Light Pixels
Extra Light Pixels
Percentage
2020
2021
2022
Pixel Distribution of WIP Human Scored Light Nuts
Amber Pixels
Light Amber Pixels
Light Pixels
Extra Light Pixels
Percentage
2020
2021
2022
Pixel Distribution of WIP Human Scored Light Amber Nuts
Amber Pixels
Light Amber Pixels
Light Pixels
Extra Light Pixels
Percentage
2020
2021
2022
Pixel Distribution of WIP Human Scored Amber Nuts
Amber Pixels
Light Amber Pixels
Light Pixels
Extra Light Pixels
Percentage
2020
2021
2022
