# Supplementary material for: Comparison of Mask-R-CNN and Thresholding-Based Segmentation for High-Throughput Phenotyping of Walnut Kernel Color
Source: Plants (Basel). 2025 Oct 31;14(21):3335. doi: 10.3390/plants14213335 (PMC12610562; doi:10.3390/plants14213335)
Supplement: Supplementary file 1 [file plants-14-03335-s001.zip › plants-3922229-supplementary_rev1/Figure S5.pdf]

THE VISUAL AIDS ON THE FOLLOWING PAGES ARE FOR REFERENCE ONLY AND NOT INTENDED FOR OFFICIAL USE.

TO PURCHASE OFFICIAL VISUAL AIDS PLEASE CONTACT THE SPECIALTY CROPS INSPECTION DIVISION'S EQUIPMENT AND FORMS DEPOT

USDA, AMS, FVP, SCI
831 MITTEN ROAD, ROOM 200
BURLINGAME, CA 94010
PHONE: 650-552-9073
FAX: 650-552-9147

EQUIPMENT CATALOG

UNITED STATES DEPARTMENT OF AGRICULTURE
WALNUT COLOR CHART

(For classifying walnut kernels in connection with the United States Standards for Grades of Shelled Walnuts and the United Standes Standars for Grades of Walnuts in the Shell)

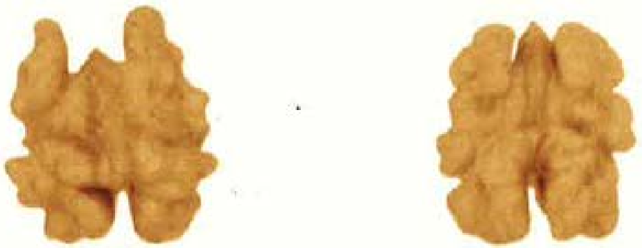

DARKEST COLOR PERMITTED IN "EXTRA LIGHT" CLASSIFICATION

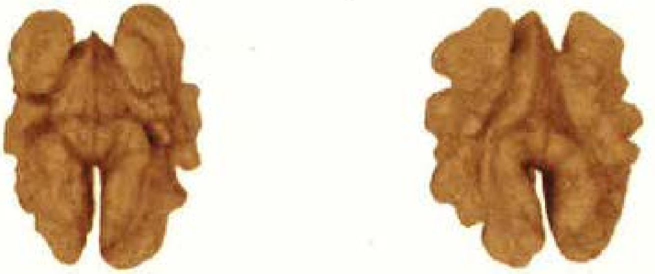

DARKEST COLOR PERMITTED IN "LIGHT" CLASSIFICATION

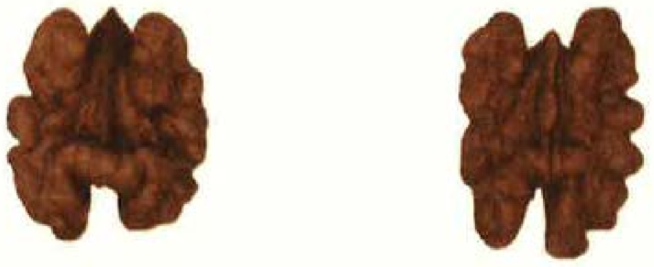

DARKEST COLOR PERMITTED IN "LIGHT AMBER" CLASSIFICATION

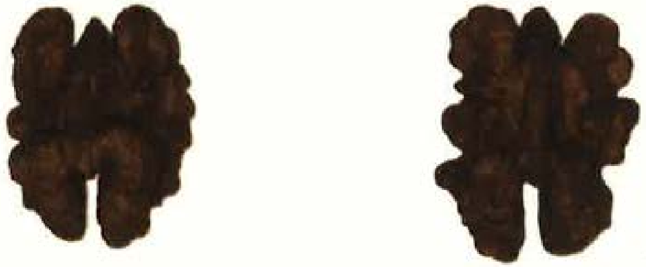

DARKEST COLOR PERMITTED IN "AMBER" CLASSIFICATION

2018
