# Supplementary figures and images for: Comparison of Mask-R-CNN and Thresholding-Based Segmentation for High-Throughput Phenotyping of Walnut Kernel Color
Source: Plants (Basel). 2025 Oct 31;14(21):3335. doi: 10.3390/plants14213335 (PMC12610562; doi:10.3390/plants14213335)

# Colorscore vs Median L\*

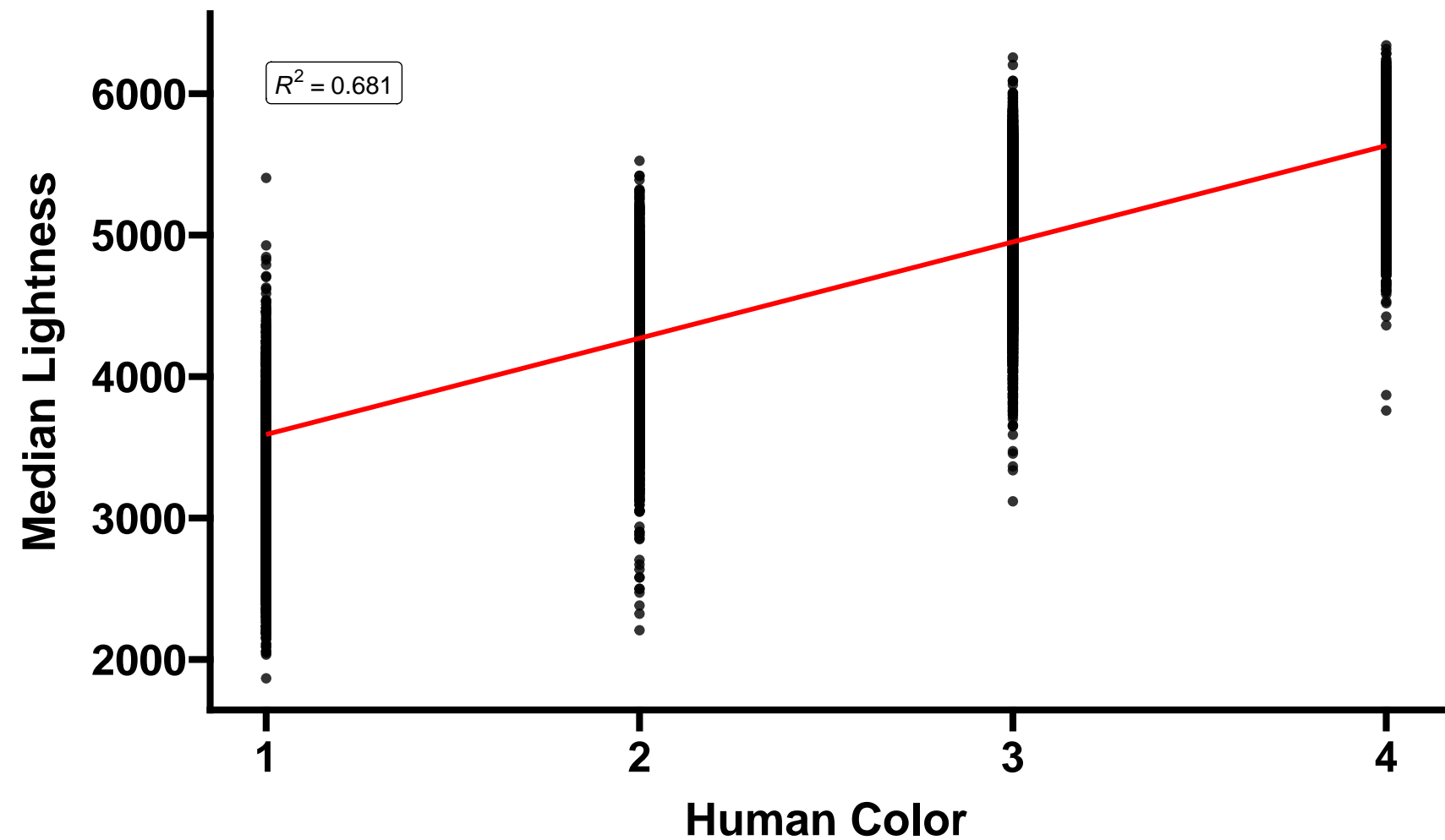

Supplement: Supplementary file 1 [file plants-14-03335-s001.zip › plants-3922229-supplementary_rev1/Figure S1.pdf]

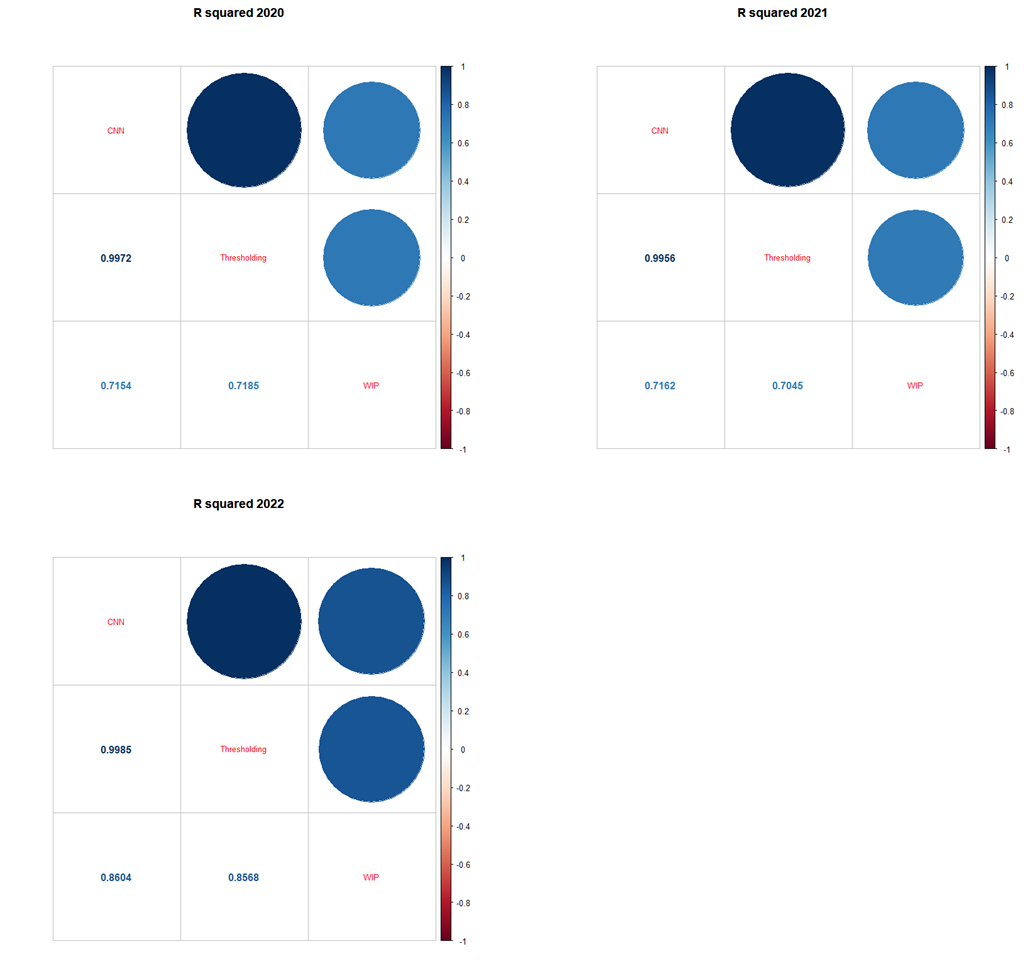

Supplement: Supplementary file 1 [file plants-14-03335-s001.zip › plants-3922229-supplementary_rev1/Figure S3.png]

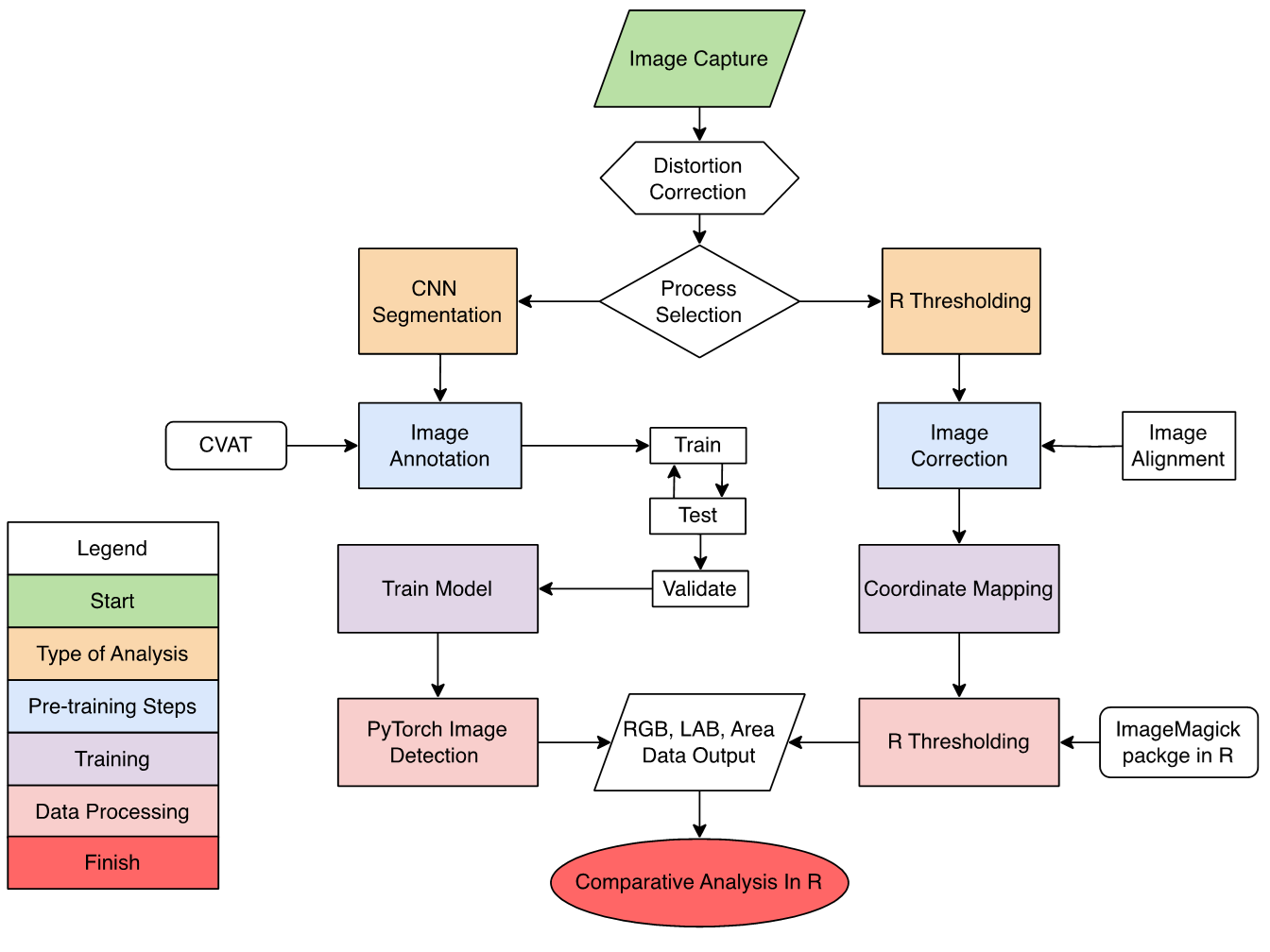

Supplement: Supplementary file 1 [file plants-14-03335-s001.zip › plants-3922229-supplementary_rev1/Figure S4.png]
